# Similarities and differences in the neural representations of abstract concepts across English and Mandarin

**DOI:** 10.1002/hbm.25844

**Published:** 2022-03-28

**Authors:** Robert Vargas, Marcel Adam Just

**Affiliations:** ^1^ Department of Psychology Carnegie Mellon University Pittsburgh Pennsylvania USA

**Keywords:** abstract concepts, cross language, fMRI, MVPA, semantic

## Abstract

Recent research suggests there is a neural organization for representing abstract concepts that is common across English speakers. To investigate the possible role of language on the representation of abstract concepts, multivariate pattern analytic (MVPA) techniques were applied to fMRI data to compare the neural representations of 28 individual abstract concepts between native English and Mandarin speakers. Factor analyses of the activation patterns of the 28 abstract concepts from both languages characterized this commonality in terms of a set of four underlying neurosemantic dimensions, indicating the degree to which a concept is *verbally represented*, *internal* to the person, contains *social content*, and is *rule‐based*. These common semantic dimensions (factors) underlying the 28 concepts provided a sufficient basis for reliably identifying the individual abstract concepts from their neural signature in the other language with a mean rank accuracy of 0.65 (*p* < .001). Although the neural dimensions used for representing abstract concepts are common across languages, differences in the meaning of some individual concepts can be accommodated in terms of differential salience of particular dimensions. These semantic dimensions constitute a set of neurocognitive resources for abstract concept representations within a larger set of regions responsible for general semantic processing.

## Graphical abstract

To investigate the possible roles of language and cultural influences on the representation of abstract concepts, multivariate pattern analytic (MVPA) techniques were applied to fMRI data to compare the neural representations of 28 individual abstract concepts between native English and Mandarin speakers. A factor analysis of the activation patterns of the 28 abstract concepts from both languages characterized this commonality in terms of a set of four underlying neurosemantic dimensions, indicating the degree a concept is verbally represented, internal to the person, contains social content, and is rule‐based. Although the neural dimensions used for representing abstract concepts are common across languages, differences in the meaning of some individual concepts can be accommodated in terms of differential salience of particular dimensions.

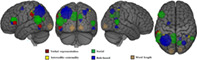

## 1 INTRODUCTION

Although the neural representations of concepts are generally similar across speakers of the same language, the extent of this similarity across languages has yet to be measured. When the concept corresponds to a concrete entity, such as an *apple*, the common basis in large part consists of the perceptual and physical properties of the referent (Just, Cherkassky, Aryal, & Mitchell, 2010). Recent studies using multivariate pattern analyses (MVPA) and machine learning techniques have reported cross‐language decoding of fMRI signatures, namely, across English and Portuguese nouns, (Buchweitz, Shinkareva, Mason, Mitchell, & Just, 2012), across English, Portuguese, and Mandarin sentences (Yang, Wang, Bailer, Cherkassky, & Just, 2017a, 2017b) as well as English, Mandarin, and Farsi stories (Dehghani et al., 2017).

The shared representational basis of abstract concepts such as *ethics* and *causality* are more difficult to identify. Given that abstract concepts do not often reflect a shared experience of the physical world, require schooling to acquire (Mason & Just, 2016), and are built on existing conceptual knowledge, there is reason to question the degree of commonality across languages in the meaning representations underlying abstract concept knowledge. Some theories have suggested that the psychological representations of abstract concepts, such as *time*, are dependent on cultural and language differences (Fuhrman et al., 2011; Lai & Boroditsky, 2013) while other theories suggest that there are culturally‐invariant neural activation patterns for concepts across brain regions (Han & Northoff, 2008). Although abstract concepts have been shown to be represented similarly across speakers within a given language (Vargas & Just, 2020), it has yet to be measured whether or not this common representation extends across languages.

Among English speakers, the neural activation patterns for abstract concepts have been shown to be underpinned by a set of three neurosemantic dimensions, namely the degree to which a concept is verbally represented; whether a concept uses the self as an internal reference; and whether the concept contains social content. Furthermore, in English, the neural representation of abstract concepts has been shown to involve regions associated with motor and visuospatial functioning (Dreyer & Pulvermüller, 2018; Harpaintner, Sim, Trumpp, Ulrich, & Kiefer, 2020). Other research has supported the emphasis of verbal and linguistic‐based processing of abstract concepts in Mandarin speakers (Wang et al., 2018). The current study compared the neural representations of the same abstract concepts in English and Mandarin to illuminate commonalities and possible differences between languages in the representation of abstract concepts.

This study had two main aims: First, to test whether a shared set of semantic dimensions underlie the neural activation patterns of abstract concepts across English and Mandarin speakers and to determine how well the observed organization of abstract concepts along these dimensions corresponds to behavioral judgments of concept meaning. Second, to identify differences in the representation of individual concepts despite a common underlying structure. Taken together, this study aims to determine whether there is a common neural basis for representing abstract concept information across languages while providing a framework for identifying language‐specific differences in the meaning of individual abstract concepts.

## 2 MATERIALS AND METHODS

### 2.1 Participants

Ten right‐handed native Mandarin speaking adults (age range from 18 to 26, *M* = 20.2; six females) and 10 right‐handed native English‐speaking adults (sample previously reported in Vargas & Just, 2020) age range from 20 to 38, *M* = 25.9; seven females;) from the Carnegie Mellon community participated in a 45‐min fMRI scanning session. To mitigate cross‐cultural familiarity, the group of native Mandarin speakers included only those who had spent less than 1 year living outside of the Peoples Republic of China. Informed consent was obtained from all participants in accordance with the Carnegie Mellon Institutional Review Board. Data from two Mandarin speakers and one English speaker were excluded due to the participant falling asleep during the scan. An additional Mandarin speaking participant's data was excluded due to their misunderstanding of instructions, resulting in data analysis of seven Mandarin speakers and nine English speakers.

### 2.2 Experimental paradigm

For both language groups, the stimuli were 28 words referring to abstract concepts distributed among seven categories. Although the category labels were never mentioned nor presented to participants, they are listed here in parentheses for expository purposes, preceding the actual stimuli: (***social***): *gossip*, *intimidation*, *forgiveness*, and *compliment*; (***emotion***): *happiness*, *sadness*, *anger*, and *pride*; (***law***): *contract*, *ethics*, *crime*, and *exoneration*; (***metaphysics***): *causality*, *consciousness*, *truth*, and *necessity*; (***religiosity***): *deity*, *spirituality*, *sacrilege*, and *faith*; (***mathematics***): *subtraction*, *equality*, *probability*, and *multiplication*; (***scientific***): *gravity*, *force*, *heat*, and *acceleration*. The set of concepts was translated from English to Mandarin by two independent native Mandarin speakers and then back‐translated to English by a separate independent Mandarin speaker. The translations were then verified by a fourth independent Mandarin–English bilingual to ensure the meaning best matches the original English concept (see Table 1 for Mandarin translations).

**TABLE 1:** Table of all 28 abstract concepts stimuli presented to English and Mandarin speaking participants

Math	Scientific	Social	Emotion	Law	Metaphysical	Religiosity
Subtraction (减法)	Gravity (引力)	Gossip (绯闻)	Happiness (幸福)	Contract (合同)	Causality (因果关系)	Deity (神明)
Equality (相等)	Force (力)	Intimidation (恐吓)	Sadness (悲伤)	Ethics (道德)	Consciousness (意识)	Spirituality (灵性)
Probability (概率)	Heat (热能)	Forgiveness (谅解)	Anger (愤怒)	Crime (罪行)	Truth (真理)	Sacrilege (亵渎)
Multiplication (乘法)	Acceleration (加速度)	Compliment (赞美)	Pride (自豪)	Exoneration (免罪)	Necessity (必要性)	Faith (信仰)

*Note*: Stimuli were presented in the participant's native languages.

Concept abstractness ratings were compared across the languages. English abstractness ratings were obtained from the Brysbaert, Warriner, and Kuperman (2014) database while Mandarin ratings were obtained from MELD‐SCH (Xu & Li, 2020). Because the concepts in the MELD‐SCH were limited to words with two characters, abstractness comparisons were restricted to the 18 concepts present in both databases (18 of 28 concepts), *r*(16) = .64, *p* < .01. Word frequencies were compared across languages using English (Brysbaert et al., 2014) and Mandarin (Cai & Brysbaert, 2010) word frequency databases. The correlation comparing the word frequencies of the concepts across languages was *r*(25) = .3, *p* = .12, indicating some minor differences in word frequency across languages.

Prior to the scanning session, participants were presented with a list of the 28 concepts and asked to write down three prominent properties of the concept's meaning. Possible properties included synonyms, definitions, or experiences associated with the concept intended to guide participants to mentally evoke a consistent representation for each concept. Participants were instructed to write properties that came to mind quickly and naturally.

There was a total of six presentation blocks of the same 28 stimulus concepts (using different random permutation orders in the different presentations) in the scanning session, distributed between three runs (two blocks per run) to allow participants a brief rest between runs. A 17‐s “X” was presented at the beginning of each block (two per run) to use as a baseline measure of neural activity. The set of 28 stimuli was presented six times to provide multiple datasets for training and testing the machine learning classifier in its cross‐validation protocol. Prior to the scan, participants briefly practiced the experimental paradigm in a mock MRI scanner while receiving head‐motion feedback to minimize movement.

On each trial, participants were visually presented with the stimulus word concept in their native language for 3 s and were asked to think about the properties associated with that concept. Following this 3 s period, participants were instructed to clear their mind over the course of 7 s while watching a blue ellipse shrink to nonexistence, to allow the hemodynamic response to approach baseline before the next concept appeared. The shrinking ellipse provided a visual fixation target and conveyed the progress through the 7 s interstimulus interval.

### 2.3 fMRI parameterization and image processing

Functional images were acquired on a Siemens Verio 3.0T scanner and a 32‐channel phased‐array head coil (Siemens Medical Solutions, Erlangen, Germany) at the Scientific Imaging and Brain Research facility (SIBR) at Carnegie Mellon. Scans were acquired using a gradient‐echo echo‐planar imagining pulse sequence (TR = 1,000 ms, TE = 25 ms, and a 60̊° flip angle); each volume contained 20 5‐mm thick AC‐PC aligned slices (1‐mm gap between slices). The acquisition matrix was 64 × 64 with 3.125 × 3.125 × 5‐mm voxels. SPM8 (http://www.fil.ion.ucl.ac.uk/spm/) was used to correct for head motion and normalize to the Montreal Neurological Institute template. The percent signal change (PSC) relative to the fixation condition was computed at each gray matter voxel for each stimulus presentation (the PSC data was converted to *z*‐scores).

The main measure of activation evoked by a concept consisted of the voxel activation levels acquired around the peak of the hemodynamic BOLD response, namely the mean of four brain images acquired once per second (i.e., a TR of 1,000) within a 4 s window, offset 5 s from the stimulus onset (i.e., images 5–8). Mean PSCs were normalized across voxels for each trial (MPSC). Previous studies have reported that the mean activation across these four images (as opposed to a GLM measure) yields a high classification accuracy obtained by a classifier that relates the activation pattern to the concept (Bauer & Just, 2017; Just et al., 2010; Mason & Just, 2016).

### 2.4 Voxel stability

The analysis focused on the most stable voxels, those whose activation levels were similarly modulated by the set of 28 abstract concepts each time the set was presented. This property selects voxels whose activation levels constitute neural signatures of a set of concepts (Bauer & Just, 2017; Just et al., 2010, 2017; Kassam, Markey, Cherkassky, Loewenstein, & Just, 2013; Mason & Just, 2016; Mason & Just, 2020; Yang et al., 2017a, 2017b). Thus, a voxel with high stability is one that has a stable tuning curve over the set of stimuli. A voxel's stability was computed as the mean pairwise correlation of its 28 MPSC activation levels (for the 28 abstract concepts) across all pairwise combinations of the presentation blocks in the training data. Stable voxels were used as features in classification and factor analyses. The stable voxels selected in the training data for classification are then used in the test set. The 120 most stable voxels in the whole brain were used as features for classification. This approximate number of voxels has been shown to reliably capture meaningful information in the neural representation of individual concepts (Just et al., 2010; Mason & Just, 2016). To demonstrate that the results and conclusions are not particularly sensitive to variations in the number of features, the classification analysis was repeated varying the number of stable voxels used from 20 to 10,000 (in 20 voxel increments); the peak classification accuracy occurred between 120 and 180 stable voxels. The mean classification accuracy gradually decreased with the inclusion of additional stable voxels beyond 180. To be consistent with previous studies, 120 stable voxels were used as features.

### 2.5 Within participant classification

The data were analyzed using various classification approaches, each informing a different aspect of the underlying concept representations. Within participant concept classification captures participant‐specific reliability as well as idiosyncrasies in concept representations. High accuracies in the within participant classification analyses suggest individual participants were able to think about a specific concept consistently and distinctly, making them identifiable by the classifier. A Gaussian Naïve Bayes (GNB) classifier was trained to decode the 28 concepts, based on its training on an independent subset of the activation data from four of the six presentations and it was tested on the mean of the two left‐out presentations. This cross‐validation procedure was followed in 15 (six choose two) folds. The features used by the classifier consisted of the activation levels of the 120 most stable voxels in the training set from anywhere in the whole brain. The classifier's mean normalized rank accuracy was used to assess decoding accuracy (i.e., the mean over folds of the normalized rank of the correct response in a probability‐ranked list of all 28 alternatives, where chance level is 0.5). Chance performance was determined using a 10,000‐iteration permutation test on each participant separately for each concept‐level prediction.

### 2.6 Between participant, within language classification

Between participant within‐language classification quantifies the commonalities of the neural representations across participants of the same language. For each language group separately, a GNB classifier was trained on the neural signatures of the concepts from all but one participant and tested on the left‐out participant's data. The mean rank accuracy was computed across the resulting nine folds for the English group and seven folds for the Mandarin group. Chance performance was determined using a 10,000‐iteration permutation test. The voxels used in the classification across participants were those with the highest stability across participants from that participant's language group. To compute the cross‐participant stability of voxels, the MPSC data was first averaged across all presentations for each participant, and then the mean pairwise correlation of a voxel's 28 MPSC activation levels (for the 28 abstract concepts) was computed between all pairs of the remaining participants in the training data. The 120 most stable voxels (i.e., those with the highest mean pairwise correlation) from the whole brain across the training participants (eight for the English group, six for the Mandarin group) were selected as features for the classifier. The methods for the cross‐language classification, which was based on the factor locations, are described below after the factor analyses.

### 2.7 Factor analysis

To uncover the semantic dimensions underlying the representations of the 28 abstract concepts, a two‐level factor analysis was computed based on the combined data from the participants of both languages; a factor analysis was first applied to the data of individual participants and then the second factor analysis used the factor scores from the first level as input (using a procedure described in detail in Just, Cherkassky, Buchweitz, Keller, & Mitchell, 2014). The factor analysis of the English‐specific activation data was previously reported and used similar methods (Vargas & Just, 2020). The factor analysis of the Mandarin‐specific data followed the same procedure with the exception that 6‐s‐level factors were extracted instead of five. The factor analysis was implemented using a principal factor analytic algorithm in MATLAB (R2011a; version 7.12; The MathWorks, Natick, MA).

The inclusion of brain regions in the combined‐language second level factor analysis was based on broad AAL (Automated Anatomical Labeling) regions containing voxels that met three criteria: the voxels had to: (1) be stable in the cross‐participant stability map; (2) have factor loadings above a threshold of ≥0.4; and (3) form clusters of at least 15 contiguous voxels. Spheres were then generated using the centroids of these clusters. The data from all 16 participants (seven Mandarin and nine English) were analyzed to identify interpretable factors. As described in Vargas and Just (2020), an initial map of the union of 800 stable voxels from each language was generated. This map was then parcellated using AAL (Tzourio‐Mazoyer et al., 2002). The parcellated map was then used to identify AAL‐defined regions with large numbers of stable voxels relative to the total number of voxels in the AAL region. Then, the input to the first‐level factor analysis (performed within each participant) consisted of the mean activation levels of the most stable voxels in each of the contributing AAL regions. The total number of voxels used in this factor analysis was 410, similar to the number used in previous studies (Kassam et al., 2013; Vargas & Just, 2020). The 410 voxels were selected with the number per AAL‐defined ROI based on the numerosity of the ROI's stable voxels in the initial map: 40 voxels from left inferior frontal gyrus (LIFG); 30 voxels from left posterior cingulate cortex; 60 voxels from frontal cortex bilaterally; 60 voxels from occipital cortex bilaterally; 60 voxels from temporal cortex bilaterally; and 160 voxels from parietal cortex bilaterally. Because the results have been shown to be insensitive to minor variations in the data analysis parameters, the same parameter values were used in this study as in Vargas and Just (2020).

To assess the dependency of the analyses on the choice of particular parameter values, the combined‐language second‐level factor analyses were computed with systematic variation of several parameters, namely the number of input voxels, number of first level factors, and number of second level factors. The effects of these variations were evaluated by correlating the factor‐scores from the second‐level dimensions across the variations and comparing the locations of the voxel clusters with high factor loadings across the variations. The effects of these variations were found to be minor, so the parameter values used in the analysis of this study were the same as those used in the previous study of these concepts (Vargas & Just 2020).

This first‐level factor analysis was performed on all 16 participants individually, extracting seven factors for each subject, resulting in a total of 112 vectors of factor scores. A voxel was determined to belong to a factor if its factor loading exceeded a threshold 0.4 (a typical value for a factor loading threshold). This same threshold was used in previous studies that characterized brain locations identified through factor analysis (Just et al., 2010, 2014; Mason & Just, 2016). To eliminate isolated single voxels, the factor‐loading voxels were required to form clusters containing a minimum of 15 voxels. Spheres for each factor were generated based on the centroids of clusters and extend to account for minor inter‐participant variations in specific voxel locations for that factor.

The goal of the first‐level factor analyses was to partition the set of input voxels into subsets that responded similarly across the set of abstract concepts, specifying seven factors. This analysis produced factor scores for the 28 concepts, for each of the seven factors, for each of the 16 participants. The 16 participants' seven sets of factor scores were concatenated and used as input into the second, group‐level factor analysis (a total of 112 sets of 28 factor scores) to further reduce the dimensionality to six dimensions and to seek consistency across participants and languages. To evaluate the robustness of the factor results, analyses were computed with varying number of input voxels and factors. Although there were minor variations in the scores of individual concepts, the overall factor interpretation and factor scores for concepts remained generally unchanged.

To confirm there is a common neural basis across languages, a factor analysis was computed on both languages separately and the factor scores were correlated between languages for each identifiable dimension. The correlations for the second‐level factor scores across languages for each identifiable dimension are as follows: Verbal representation: *r* = .55, *p* < .01; rule‐based: *r* = .45, *p* < .05; social content: *r* = .42, *p* < .05; externality–internality: *r* = .32, *p* < .1; word length: *r* = .20, n.s. Notably, the previously unexplainable factor described in Vargas and Just (2020) was reliably correlated with the newly identified rule‐based factor in the Mandarin group. Additionally, the lack of correlation between the low correlation of the word length factor scores across languages reflects language‐specific orthographic differences.

Regions for the factor analysis of both languages combined were selected based on their being populated by stable voxels. Whole‐brain voxel‐wise stability was computed for each participant separately and averaged across participants. This method allows for a spatially stable common set of voxels to be identified. The interpretation of each individual factor was largely based on the distribution of the corresponding factor scores across the 28 concepts (particularly the nature of the items at the two extremes of the factor scores) and based to some degree on previous findings that associated particular processes with the factor locations. Moreover, converging evidence for the factor interpretations was provided by the correlation between the factor scores and independent participant ratings of the items with respect to the factor as interpreted.

#### 2.7.1 Behavioral rating of semantic dimensions

To obtain converging evidence for the factor interpretations, an independent group of 20 participants (10 native English speakers and 10 native Mandarin speakers) were asked to rate each stimulus concept on a scale from 1 to 7 with respect to its salience to the dimensions as they were interpreted here (e.g., the degree to which a concept, such as *ethics*, was verbally vs. perceptually based). These ratings of the concepts along each of its dimensions were then used as independent variables in a multiple regression model to predict the activation pattern of a concept in the factor locations (Just et al., 2010; Vargas & Just, 2020).

The correlations between the behavioral ratings of English and Mandarin participants for the 28 concepts on each dimension were as follows: Verbal representation, *r* = .67, *p* < .001; externality–internality, *r* = .93, *p* < .001; rule‐based, *r* = .94, *p* < .001; social content, *r* = .9, *p* < .001. Given the highly reliable correlation between English and Mandarin behavioral ratings, averaged ratings were used as input to the regression model.

#### 2.7.2 Predictive modeling

To evaluate the how well the factor interpretations fit the activation data, a predictive modeling procedure was used to assess whether the activation pattern of an individual concept could be predicted, based on the mapping between behavioral ratings of all the other concepts in the set (i.e., leaving out the to‐be predicted item) with respect to the factor interpretations and their activation patterns. Accurate predictions would provide face validity for the factor interpretations. Activation predictions for each concept were made by developing a separate regression model for each participant to predict a left‐out concept's activation pattern, based on the model weights from the remaining 27 concepts. The factor locations used were obtained from factor analyses based on all participants except for the one being predicted. The mean prediction accuracies for the 28 concepts were then averaged across participants. A prediction's accuracy was assessed by computing the Euclidean distance between the activation pattern predicted by the model and the observed activation data, relative to the distance to the representations of the other 27 concepts. The normalized rank of the distance between the predicted and test images (among the 28 distances) was the measure of prediction accuracy. Significance was computed using a permutation test. The results of the predicted images with correct labels were compared against the distribution of rank accuracies of predicted images with random labels for 10,000 random permutations.

### 2.8 Factor‐based cross‐language classification

Cross‐language factor‐based classification quantifies the commonality of representation across languages based on the semantic dimensions underlying the concept representations. To test whether the factors (or dimensions) are sufficient for identifying the neural signatures of individual abstract concepts across languages, a GNB classifier was trained on the neural signatures from all participants from one language and was tested individually on each of the participants from the other language. The data consisted of the mean MPSC values of each concept across repetitions for each participant in the factor locations of the five interpretable factors in the factor analysis including both languages. A classifier was trained on the data of all nine native English speakers and was tested on each of the seven native Mandarin speakers and vice versa. The 28 rank accuracies from each participant in the test language were then averaged. There were minimal differences in accuracies between the two classifiers *t*(27) = 0.01, n.s., so the accuracies of the two classifiers were averaged. Above‐chance performance at *p* < .01 is 0.56 for concept‐level predictions as determined using a 10,000‐iteration permutation test.

## 3 RESULTS

### 3.1 Systematicity and commonality of abstract concept representations within and across languages

#### 3.1.1 Within‐participant classification in the two languages

The individual 28 abstract concepts were reliably identified from their multi‐voxel neural signatures within each language by a classifier. This mean classification accuracy for native English participants, 0.83, was reliably above chance (range = 0.76–0.94, *p* < .001; mean cutoff for *p* < .001 = 0.60; SD = 0.003) as was that of the seven native Mandarin participants (mean = 0.77; range = 0.66–0.84). Although the concepts of all participants in both groups were identifiable, a *t*‐test comparing the within‐participant classification accuracies of the 28 concepts across languages indicated that the classification accuracy was reliably higher in the English participants, *t*(27) = 6.70, *p* < .001.

Although the concepts differ in their overall identifiability between the two language groups, these results indicate that these abstract concepts have distinctive neural signatures in both languages that can be characterized by the multi‐voxel activation pattern captured by the classifier.

#### 3.1.2 Commonality of the concept representations across speakers of the same language

A between‐participant, within‐language classification was performed to determine whether these abstract concept representations were similar across speakers *within* a language group. For English speakers, when the classifier was trained on the data of all but one participant, the mean rank accuracy of the concept identification in the data from the left‐out participant was 0.74, *p <* .01, indicating that the neural signatures had a substantial amount of commonality across participants (Table 2). All 28 individual concepts were reliably classifiable between English‐speaking participants, with a range of 0.58–0.94 (*p* < .01 = 0.55).

**TABLE 2:** Commonality of concepts within and across languages as measured using concept‐level decoding rank accuracy. Dashed lines separate concept categories.

	Mandarin Between‐Participant	Mandarin Within‐Participant	English Between‐Participant	English Within‐Participant	Cross‐Language
Subtraction	0.72	0.79	0.94	0.89	0.70
Equality	0.7	0.77	0.58	0.8	0.67
Probability	0.69	0.72	0.8	0.86	0.49
Multiplication	0.91	0.81	0.73	0.92	0.82
Gravity	0.8	0.8	0.86	0.88	0.79
Force	0.84	0.84	0.84	0.87	0.83
Heat	0.63	0.7	0.78	0.8	0.74
Acceleration	0.92	0.77	0.76	0.84	0.77
Gossip	0.63	0.79	0.66	0.79	0.68
Intimidation	0.72	0.82	0.75	0.83	0.73
Forgiveness	0.75	0.69	0.77	0.7	0.80
Compliment	0.81	0.72	0.67	0.81	0.58
Happiness	0.76	0.71	0.75	0.76	0.60
Sadness	0.69	0.78	0.71	0.85	0.76
Anger	0.59	0.79	0.62	0.82	0.62
Pride	0.78	0.86	0.86	0.88	0.84
Contract	0.52	0.77	0.63	0.75	0.66
Ethics	0.71	0.78	0.72	0.83	0.56
Crime	0.66	0.72	0.76	0.8	0.69
Exoneration	0.63	0.77	0.76	0.83	0.41
Causality	0.91	0.89	0.8	0.89	0.62
Consciousness	0.66	0.77	0.79	0.84	0.53
Truth	0.69	0.77	0.62	0.86	0.55
Necessity	0.9	0.82	0.78	0.77	0.57
Deity	0.77	0.69	0.59	0.78	0.60
Spirituality	0.57	0.72	0.79	0.82	0.46
Sacrilege	0.61	0.73	0.62	0.83	0.52
Faith	0.81	0.72	0.78	0.81	0.61
**Mean**	**0.73**	**0.77**	**0.74**	**0.83**	**0.65**

*Note*: Dashed lines separate concept categories.

For Mandarin speakers, when the classifier was trained on the data of all but one participant, the mean rank accuracy of the concept classification in the test data from the left‐out participant was 0.73, *p <* .01 (Table 2). All 28 individual concepts were reliably classifiable between Mandarin‐speaking participants, with a range of accuracies from 0.57 to 0.92 (*p <* .01 = 0.54) except for *contract* which was classifiable only at *p* < .05.

Thus, there is a comparable degree of commonality across participants within each language group in their neural representation of the abstract concepts. Below, the underlying dimensions of the concept representations across languages are described, followed by an assessment of the commonality of the neural representations of individual concepts across languages, taking the underlying dimensions into account.

In the few cases where between‐participant decoding was more accurate than within‐participant decoding within a language, the difference might be attributable to the different way the stable voxels were selected in the two cases. The consensually chosen stable voxels in the between‐participant analysis could have reduced idiosyncratic properties in the concept representations.

#### 3.1.3 Mandarin‐specific factor analysis

**FIGURE 1:**
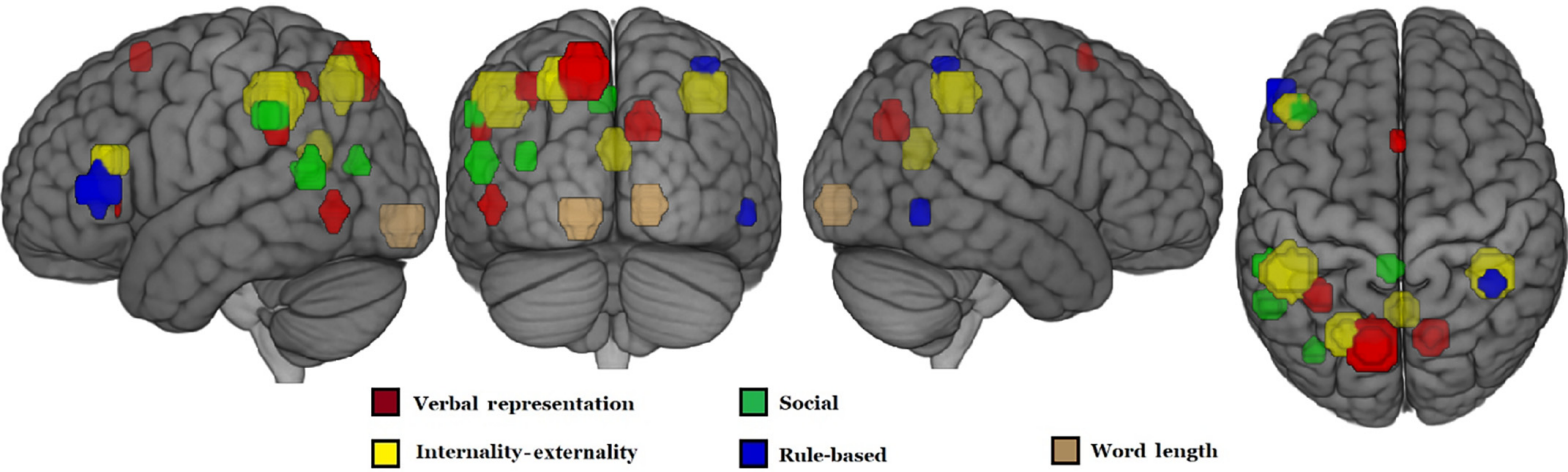
Locations for five interpretable factor dimensions from Mandarin‐specific analysis. These spheres were specified using the centroids of clusters of voxels (containing a minimum of 10 voxels) with high loadings (>0.4) on each of the factors

The Mandarin‐specific factor analysis indicated a common neurosemantic basis for the set of 28 abstract concepts across English and Mandarin, revealing five interpretable dimensions, namely: Verbal representation, social content, rule‐based, externality/internality, and word length. The concepts located at the extremes of each of these dimensions and their respective factor scores are shown in Table 3. The correlations between the Mandarin behavioral ratings and Mandarin‐only factor scores for the 28 concepts on each dimension were as follows: Verbal representation, *r* = .57, *p* < .01; externality–internality, *r* = .66, *p* < .001; rule‐based, *r* = .34, *p* = .07; social content, *r* = .27, *p* = .16. The brain locations of the voxel clusters with high loadings on the interpretable factors for the Mandarin‐specific analysis are shown as spheres in Figure 1.

**TABLE 3:** Mandarin‐only factor analysis output including: six concepts with the highest and lowest factor scores for each mapped dimension, factor locations, and correlations between factor scores and behavioral ratings

Verbal representation	Externality/internality	Rule‐based	Social content	Word length
Faith (2.17)	Subtraction (2.20)	Truth (1.48)	Intimidation (1.73)	Causality (3.23)
Spirituality (1.78)	Equality (1.84)	Acceleration (1.46)	Sadness (1.63)	Necessity (2.22)
Deity (1.24)	Gravity (1.57)	Gravity (1.03)	Equality (1.52)	Acceleration (1.61)
Compliment (1.19)	Force (1.00)	Sadness (1.02)	Contract (1.21)	Happiness (0.89)
Probability (1.02)	Causality (0.93)	Causality (0.96)	Ethics (1.06)	Exoneration (0.65)
Causality (0.98)	Contract (0.50)	Force (0.84)	Gossip (0.65)	Compliment (0.46)
Forgiveness (−1.10)	Spirituality (−0.78)	Consciousness (−1.34)	Crime (−0.78)	Sadness (−0.72)
Consciousness (−1.32)	Deity (−0.88)	Gossip (−1.39)	Happiness (−0.89)	Contract (−0.79)
Gravity (−1.38)	Forgiveness (−0.99)	Necessity (−1.43)	Force (−1.30)	Faith (−0.93)
Acceleration (−1.56)	Pride (−1.19)	Anger (−1.45)	Heat (−1.55)	Equality (−1.18)
Sadness (−1.60)	Happiness (−1.38)	Sacrilege (−2.12)	Gravity (−2.07)	Force (−1.46)
Anger (−1.76)	Sadness (−1.42)	Compliment (−2.55)	Spirituality (−2.60)	Sacrilege (−1.61)

#### 3.1.4 Combined‐language factor analysis

**FIGURE 2:**
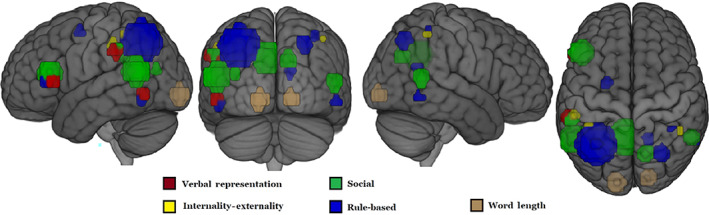
Locations for five interpretable factor dimensions from combined‐language analysis. These spheres were specified using the centroids of clusters of voxels (containing a minimum of 10 voxels) with high loadings (>0.4) on each of the factors

The commonality of neural representation for abstract concepts in the two languages was characterized by six underlying dimensions, five of them readily interpretable. Of the five interpretable dimensions, four were semantic in nature, which we have labeled: Verbal representation, internality–externality to self, rule‐based, and social content. (Each dimension is further described in the Discussion). The remaining non‐semantic dimension corresponded to the length of the printed word that named the concept. The five interpretable group‐level factors accounted for 36% of the variance in the participant‐level factors. All but one of these factors (rule‐based concepts) have been identified in a previous study of abstract concepts (Vargas & Just, 2020). The brain locations of the voxel clusters with high loadings on the interpretable factors are shown as spheres in Figure 2 (cluster centroid xyz coordinates for each factor can be found in Table S1). The concepts located at the extremes of each of these dimensions and their respective factor scores are shown in Table 4.

**TABLE 4:** The six concepts with the highest and lowest factor scores for each interpretable dimension from the combined‐language factor analysis

Verbal representation	Externality/internality	Rule‐based	Social content	Word length
Ethics (1.23)	Causality (1.79)	Multiplication (2.00)	Intimidation (1.40)	Causality (1.88)
Spirituality (1.19)	Gravity (1.53)	Subtraction (1.97)	Pride (1.33)	Acceleration (1.73)
Faith (1.19)	Sacrilege (1.29)	Probability (1.61)	Gossip (1.29)	Happiness (1.43)
Sacrilege (1.06)	Equality (1.06)	Acceleration (0.85)	Forgiveness (1.21)	Probability (1.19)
Necessity (0.89)	Subtraction (0.99)	Ethics (0.82)	Exoneration (1.11)	Compliment (0.99)
Exoneration (0.82)	Crime (0.67)	Contract (0.74)	Anger (0.72)	Necessity (0.86)
Sadness (−0.73)	Anger (−0.90)	Crime (−0.77)	Subtraction (−0.63)	Truth (−0.90)
Happiness (−1.19)	Forgiveness (−0.93)	Exoneration (−0.93)	Happiness (−0.65)	Ethics (−1.05)
Acceleration (−1.67)	Pride (−1.36)	Consciousness (−1.08)	Necessity (−0.77)	Pride (−1.24)
Force (−1.73)	Spirituality (−1.47)	Sacrilege (−1.30)	Multiplication (−0.92)	Faith (−1.52)
Gravity (−2.06)	Happiness (−1.82)	Anger (−1.36)	Heat (−1.77)	Crime (−1.59)
Heat (−2.12)	Sadness (−2.06)	Compliment (−1.70)	Spirituality (−3.15)	Force (−2.33)

Behavioral ratings of each concept reflecting the saliency of each dimension (as it had been interpreted) were used as independent variables in a linear regression model that predicted the activation level of each concept in the factor locations. The mean rank accuracy of predictions for left‐out concepts, averaged first over concepts and then over participants, was 0.73, *p* < .001. Performing the predictive modeling analysis while excluding the word length dimension resulted in a mean classification accuracy of 0.72, *p* < .001, which was not significantly different from the accuracy when word length was included, *t*(27) = 1.80, n.s.

The correlation for a given dimension between the factor scores of the 28 concepts and their behavioral ratings were reliable for all semantic dimensions for both languages. The correlations between ratings and factor scores from the language‐specific factor analyses for each semantic dimension are as follows: the externality dimension had an *r* = .63, *p* < .001 for Mandarin and *r* = .70, *p* < .001 for English; the social dimension had an *r* = .52 *p* < .01 for Mandarin and *r* = .46, *p* < .05 for English; and the rule‐based dimension had an *r* = .40, *p* < .05 for Mandarin and *r* = .39, *p* < .05 for English. The similarity between languages in the correlations between factor scores and mean behavioral ratings for the Verbal dimension: in the case of the English ratings, it was *r* = .82, *p* < .001, and for the Mandarin ratings, it was *r* = .42, *p* < .05. These significant correlations between the behavioral ratings and factor scores indicate convergent validity for the interpretations of the semantic dimension for both English and Mandarin samples.

### 3.2 Common representation supported by cross‐language classification

To assess the similarity of individual concept representations across languages based on the underlying factors, a classifier was trained on one language to predict individual concepts in the other language. Cross‐language classification of individual concepts resulted in a mean rank accuracy (averaged over concepts, direction of decoding, and participants) of 0.65, *p* < .001. (The right‐most column in Table 2 shows the accuracies for individual concepts.) When individual concepts were averaged within categories, the categories with the highest accuracies were mathematics (0.67), scientific (0.78), emotion (0.70), and social (0.70). When the features for the between participant cross‐language classification were defined independently of the factors, using the union of 120 stable voxels from each language regardless of their association with any of the factors, the mean accuracy was 0.69, *p* < .01. The classification accuracy was only slightly lower (0.65 vs. 0.69) when computed using only stable voxels associated with factors, indicating how well a factor‐based account of the data accounts for the similarity between languages in their neural representations of abstract concepts.

### 3.3 Differences in the neural representation of concepts across languages

Although a common set of dimensions was identified (as indicated by the reliable correlations of factor scores across the two language‐specific factor analyses in Table 5), the distribution of the items along corresponding factors was similar but not identical (Table 3 and table 1 from Vargas & Just, 2020).

**TABLE 5:** Correlation matrix of factor scores across English (rows) and Mandarin (columns) factor analyses for each semantic dimension

English‐by‐Mandarin	Verbal representation	Word length	Externality/internality	Rule‐based	Social content
Verbal representation	0.55 (*p* < .01)	−0.05	−0.29	−0.38	0.31
Word length	0.01	0.20 (n.s)	−0.33	−0.11	0.14
Externality/internality	0.42	0.08	0.32 (*p* < .1)	−0.14	0.13
Rule‐based	0.12	0.08	0.35	0.45 (*p* < .05)	0.40
Social content	−0.42	−0.23	−0.28	0.05	0.42 (*p* < .05)

*Note*: The variance each dimension accounted for varied across languages but were aligned here for easier comparison.

Abbreviation: n.s, not significant

Independently collected behavioral ratings provided converging evidence that a few individual abstract concepts are represented somewhat differently along the verbal representation dimension. English speakers rated emotions (e.g., *happiness* and *anger*), social concepts (e.g., *intimidation* and *compliment*) and spiritual concepts (e.g., *deity* and *sacrilege*) as being more verbally represented than did Mandarin speakers. Additionally, Mandarin speakers rated mathematical concepts (e.g., *subtraction* and *multiplication*) and scientific concepts (e.g., *heat* and *acceleration*) as more verbally represented than did English speakers.

Qualitative descriptions of the concept properties that participants reported suggest polysemous words such as *equality* were represented somewhat differently across languages. English speakers tended to interpret the concept of *equality* partly in the context of social equality while Mandarin speakers tended to interpret *equality* in terms of its mathematical meaning. These results suggest that the difference between languages is not in brain function but in the meanings of the “translation equivalents” of the polysemous word *equality* in the two languages. These differences could be due in part to differences in relative frequency or prominence of the two senses of the word in the two languages.

## 4 DISCUSSION

### 4.1 Overview

The results of this study suggest that a common neural infrastructure exists for representing abstract concepts across English and Mandarin. Factor analyses using activation data from both languages revealed four semantically interpretable dimensions, *verbal representation*, *internality–externality*, *social content*, and *rule‐based representation* underlying the activation patterns of 28 abstract concepts for both languages. A secondary finding was that although the neural regions or systems associated with these representations are common, the representations of individual concepts sometimes differ with respect to the salience of an underlying dimension.

### 4.2 Language‐invariant semantic primitives of abstract concept representation

The four emerging neurosemantic dimensions underlying representation of abstract concepts are: *verbal representation*, *internality–externality*, *social content*, and *rule‐based representation*. The ability to think of abstract meaning draws on the ability to think of concepts in terms of other verbal concepts, to rely on the use of the self as a reference, to think in terms of social contexts, and to consider the rules that govern certain concepts. The combined‐language factors highlight possible subnetworks that are present in the general semantic network identified by Ralph, Jefferies, Patterson, and Rogers (2017). These subnetworks represent the processing of specific types of semantic information. Regardless of language, people rely on the same broad neural systems to represent abstract concept meaning.

Although previous research has found emotion processing to be involved in the processing of abstract concepts (Kassam et al., 2013; Kousta, Vigliocco, Vinson, Andrews, & Del Campo, 2011; Vigliocco et al., 2014), an emotion‐specific dimension did not emerge in our factor analyses. However, one of the factor locations of the social content dimension identified from the combined‐language factor analysis, posterior cingulate, is often associated with affective processing. The inclusion of this region could be due to the affective involvement in abstract concepts like intimidation, pride, and forgiveness for the social dimension. Vigliocco et al.'s (2014) abstract stimuli were selected on a different basis (concreteness ratings) than ours (membership in seven abstract semantic categories). Many of our abstract categories (such as science, mathematics, and metaphysics (logic) and hence their members (e.g., gravity, force, acceleration, and causality, truth, necessity) are sparsely represented in the Vigliocco stimulus set. By contrast, the Vigliocco stimulus set contains many descriptors of mental states that have a significant affective component, such as agony delirium, frenzy, and panic. It may be that an affective component plays a larger role in the representations of abstract concept representations pertaining to mental states rather than to physical or constructed worlds.

#### 4.2.1 The verbal representation dimension of abstract concepts

This dimension organizes concept representations based on their degree of association with word (verbal) representations (manifested as activation in left inferior frontal gyrus (LIFG, specifically, the triangular subregion) and disassociation with visuospatial processing and action imagery (lower activation in LLOC and LSMG) (Vargas & Just, 2020). The verbal representation dimension accounted for the most variance (9%) in the participant‐level factor representations and was the most salient dimension for all participants in both languages. Concepts such as *faith*, *spirituality*, and *ethics* anchor the verbal extreme of this dimension while concepts such as *heat*, *gravity*, and *force* anchor the other, nonverbal extreme. The concepts at the verbal extreme evoke activation in language areas, presumably because the concept evokes the thought of verbal labels for other related concepts. The presence and salience of this dimension in these data and in previous studies work (Vargas & Just, 2020; Wang, Conder, Blitzer, & Shinkareva, 2010) provide converging evidence for the prominent role of this semantic dimension in the representation of abstract concepts.

#### 4.2.2 The self‐based dimension of abstract concepts

The internality–externality dimension organizes conceptual representations based on the degree to which a concept uses the self as a reference. Concepts such as *pride*, *spirituality*, and *sadness* anchor the internal extreme of the dimension while the concepts *causality*, *gravity*, and *sacrilege* anchor the external extreme. This dimension accounted for 8% of the variance in the participant‐level factor analysis. This factor's locations include RSMG, a region shown to be related to the projection of one's own mental state onto others (Silani, Lamm, Ruff, & Singer, 2013).

#### 4.2.3 The social interaction dimension of abstract concepts

This dimension organizes abstract representations based on whether they entail a social component. The concepts *intimidation*, *pride*, *gossip*, and *forgiveness* typify the extreme of this dimension. This dimension accounts for 6.4% of the variance in the participant‐level factor analysis. The factor locations include regions associated with autobiographical information processing (posterior cingulate) and theory‐of‐mind (right temporoparietal junction), as well as the triangular region of LIFG. Although the triangular region of LIFG was a component of the social dimension in the combined‐language factor analysis, the presence of this component emanates from the Mandarin‐specific data (Figure 1). This participation of LIFG in the Social dimension only in Mandarin is an example of differential involvement of various components of meaning across languages. It is uncertain from our study what functional role LIFG is contributing to this dimension; for example, it is possible that some abstract concepts with a social component also entail an associated verbal expression that LIFG references and this LIFG role may be differentially used in Mandarin.

#### 4.2.4 The rule‐based dimension of abstract concepts

This dimension organizes concepts in terms of their being based on some set of rules or that define or are defined by specific, precise relationships between other concepts. The concepts *multiplication*, *probability*, and *ethics* typify this dimension. The factor locations are left precuneus and right supramarginal gyrus. This factor accounts for 7.1% of the variance in the participant‐level factor analysis. The regions associated with this dimension share a partial overlap in left parietal cortex with regions previously identified to be associated with algebraic and equation‐based processing (Mason & Just, 2016). The Mandarin‐specific factor analysis indicated that one of the rule‐based factor locations was in the triangular region of LIFG (Figure 1). This region is sometimes associated with semantic selection (Badre, Poldrack, Paré‐Blagoev, Insler, & Wagner, 2005), and its role in the rule‐based representations may entail reference to a verbal expression of some aspect of a rule.

#### 4.2.5 The neural representation of word length in English and Mandarin

This dimension organizes the concepts in terms of the length of the written word that names them. For English, the word length factor scores are correlated with the number of characters in a word, with *r* = .68, *p* < .001. For Mandarin concepts, the number of strokes was used as a measure of word length. There was insufficient variance in the number of characters of the set of Mandarin concepts, with 24 of the 28 concepts containing two characters (*M* = 2.04; *SD* = 0.5). For Mandarin, the word length factor scores are correlated with the number of strokes in a word, with *r* = .56, *p* < .01. Although the number of characters and the number of strokes in a word are not correlated (*r* = .2, n.s.), when normalized (converted to *z*‐scores) and averaged, the resulting relative‐word‐length measure of participants of both languages was correlated with the factor scores of this dimension at *r* = .8, *p* < .001. The region associated with this dimension (i.e., where the voxels with high loadings on this factor are located) is isolated to the occipital pole. This finding suggests the presence of a language‐common, word length dimension that captures some aspect of the early visual percept of the word.

### 4.3 Commonality of meaning for abstract concepts

The common neural organization of the concepts, as characterized by the shared underlying semantic dimensions, was sufficient to serve as a basis for reliably classifying (identifying) individual concepts using the activation signature from the other language. This cross‐language classifiability provides converging evidence for a language‐invariant semantic representation of abstract concepts across the set of 28 concepts. The high cross‐language decodability of mathematics and science concepts could be attributable to the language‐invariant algebraic expression of these concepts (Mason & Just, 2016). The high cross‐language decodability of emotion and social concepts could be attributable to a common embodied nature of these concepts.

The similarity of the factor analysis outcomes in the two languages indicates a common underlying neurocognitive infrastructure for processing abstract concepts. This semantic resource distributed across multiple brain locations constitutes a more specialized subset of regions previously identified as a general semantic network (Ralph et al., 2017). These regions were sufficient for reliably decoding individual abstract concepts across languages based on their neural representations. However, some of the underlying dimensions were differentially salient across languages.

### 4.4 Nuances in the meaning of individual abstract concepts across languages

Not all concepts were decoded equally accurately across languages; in each language, there were individual concepts that were reliably classified within language but not across language. For example, causality was highly decodable within English (0.89 accuracy) and Mandarin (0.8) but less decodable across languages (0.62). In these cases, the semantic properties (e.g., contexts or associated concepts) that were generated by participants for these concept representations could be homogenous within each language but distinct across languages.

Low cross language decodability could often be explained by a differential salience of verbal processing (as is this case for some concepts such as *consciousness*, *necessity*, or *faith*) while other concept differences could be attributable to differing senses of meaning across languages. One such example cited above is that in Mandarin, the concept, *equality*, was more strongly interpreted in its mathematical sense than social sense relative to the English interpretation. These differences could be caused by differences in how concepts like *equality* are learned or taught, or the differences may arise because of the polysemy of some of the words used to describe the abstract concept. Due to small sample sizes, it is uncertain whether cultural differences or characteristics of the two samples of participants or nonequivalence of the stimulus words' connotation or senses were responsible for the differences between languages in some of the neural representations.

In addition to occasional nuances of a difference between languages in the neural representations of individual concepts, the underlying dimensions sometimes played a larger role in one language than the other. For example, the verbal representation dimension accounted for 10% of variance in the English factor analysis but only 7% in the Mandarin analysis. Differences such as these indicate that abstract concepts in the two languages can evoke various dimensions of meaning to different degrees. Such differences could be attributable to differences in the senses or connotation of the translation‐equivalent word concepts.

## 5 CONCLUSION

Factor analyses revealed a set of common neurosemantic dimensions that constitute the basis for the representation of abstract concepts across languages: verbal representation, internality–externality, social content, and rule‐based content. The subsequent predictive modeling based on behavioral ratings of the concepts provides convergent validity for the factor interpretations. The successful cross‐language classification suggests that the underlying semantic dimensions provide a sufficient basis for decoding abstract word concepts across languages. Although the neural dimensions used for representing abstract concepts are common across languages, differences in the meaning of some individual concepts can be accommodated in terms of differential salience of particular dimensions. These semantic dimensions constitute a set of neurocognitive resources for abstract concept representation within a larger set of regions responsible for general semantic processing.

## CONFLICT OF INTEREST

The authors declare no competing interests.

## Supporting information


**Table S1** Combined‐language cluster centroid xyz coordinates for each semantic dimension.

## Data Availability

The preprocessing was conducted using toolboxes from SPM8. Machine learning analyses were constructed from functions found in Matlab R2011a as described in the Methods section. The datasets used in this project are available from either author upon request.
