# Supplementary material for: Similarities and differences in the neural representations of abstract concepts across English and Mandarin
Source: Hum Brain Mapp. 2022 Mar 28;43(10):3195–206. doi: 10.1002/hbm.25844 (PMC9188971; doi:10.1002/hbm.25844)
Supplement: Supplementary file 1 — Table S1 Combined‐language cluster centroid xyz coordinates for each semantic dimension. [file HBM-43-3195-s001.docx]

Supplemental Information

**S1.** Combined-language cluster centroid xyz coordinates for each semantic dimension.
